# Ribosomal RNA-Depletion Provides an Efficient Method for Successful Dual RNA-Seq Expression Profiling of a Marine Sponge Holobiont

**DOI:** 10.1007/s10126-022-10138-8

**Published:** 2022-07-27

**Authors:** Xueyan Xiang, Davide Poli, Bernard M. Degnan, Sandie M. Degnan

**Affiliations:** 1 School of Biological Sciences, University of Queensland, Brisbane, QLD 4072 Australia; 2 Present Address: BGI-Shenzhen, Shenzhen, 518083 China; 3 Present Address: School of Biomedical Sciences, Queensland University of Technology, Brisbane, Australia

**Keywords:** Demosponge, Holobiont, Hologenome, Porifera, Transcriptomics

## Abstract

Investigations of host-symbiont interactions can benefit enormously from a complete and reliable holobiont gene expression profiling. The most efficient way to acquire holobiont transcriptomes is to perform RNA-Seq on both host and symbionts simultaneously. However, optimal methods for capturing both host and symbiont mRNAs are still under development, particularly when the host is a eukaryote and the symbionts are bacteria or archaea. Traditionally, poly(A)-enriched libraries have been used to capture eukaryotic mRNA, but the ability of this method to adequately capture bacterial mRNAs is unclear because of the short half-life of the bacterial transcripts. Here, we address this gap in knowledge with the aim of helping others to choose an appropriate RNA-Seq approach for analysis of animal host-bacterial symbiont transcriptomes. Specifically, we compared transcriptome bias, depth and coverage achieved by two different mRNA capture and sequencing strategies applied to the marine demosponge *Amphimedon queenslandica* holobiont. Annotated genomes of the sponge host and the three most abundant bacterial symbionts, which can comprise up to 95% of the adult microbiome, are available. Importantly, this allows for transcriptomes to be accurately mapped to these genomes, and thus quantitatively assessed and compared. The two strategies that we compare here are (i) poly(A) captured mRNA-Seq (Poly(A)-RNA-Seq) and (ii) ribosomal RNA depleted RNA-Seq (rRNA-depleted-RNA-Seq). For the host sponge, we find no significant difference in transcriptomes generated by the two different mRNA capture methods. However, for the symbiont transcriptomes, we confirm the expectation that the rRNA-depleted-RNA-Seq performs much better than the Poly(A)-RNA-Seq. This comparison demonstrates that RNA-Seq by ribosomal RNA depletion is an effective and reliable method to simultaneously capture gene expression in host and symbionts and thus to analyse holobiont transcriptomes.

**Supplementary Information:**

The online version contains supplementary material available at 10.1007/s10126-022-10138-8.

## Introduction

Increasing interest in the microbes that live in association with multicellular organisms has revealed that symbioses are ubiquitous, and that plants and animals are best considered as holobionts comprising both a multicellular host and diverse microbiota (Bordenstein and Theis 2015; Rosenberg and Zilber-Rosenberg 2016). Host-associated microbes can have a critical influence on host metabolism, immunity, physiology, development and fitness (Engelstadter and Hurst 2009; Ezenwa et al. 2012; Leulier et al. 2017; McFall-Ngai et al. 2013; Pradeu 2011; Rosenberg and Zilber-Rosenberg 2016; Song et al. 2021; Yen and Barr 1971). To investigate these influences, the mechanisms of host-symbiont interactions are increasingly being explored by multi-omics approaches (Heinken and Thiele 2015; Rowland et al. 2017).

With recent significant reductions in sequencing costs, RNA-sequencing (RNA-Seq) has become a widely used method to study host-symbiont interactions and is especially valuable when used to profile host and symbiont transcriptomes simultaneously in the same biological samples. In the past decade, several studies have applied dual transcriptome analysis to reveal potential modes of cross-talk between symbiosis partners. For instance, early transcriptome analysis of the whitefly (*Bemisia tabaci*) complex revealed complementation in amino acid biosynthesis pathways between the whitefly and its bacterial endosymbiont *Candidatus Portiera aleyrodidarum* (Upadhyay et al. 2015). Dual RNA-Seq across the life cycle of the nematode *Brugia malayi* and its bacterial endosymbiont *Wolbachia* identified developmental pathways involving both nematode and bacterial genes (Grote et al. 2017). Metatranscriptome analysis contributed to the reconstruction of metabolic pathways of the sponge *Cymbastela concentrica* and its symbiotic microbes (Moitinho-Silva et al. 2017) and coral reef demosponges more broadly (Robbins et al. 2021).

However, the accurate and simultaneous analysis of the transcriptional state of both animal and bacterial partners in a symbiosis currently is not optimised for many holobiont systems. Transcriptomes generated via RNA-Seq tend to be dominated by highly abundant ribosomal (r)RNAs, making it necessary first to remove rRNA and enrich for coding sequence mRNAs. This need for rRNA removal creates challenges for studies wishing to capture both eukaryotic and bacterial mRNAs, because of differences in eukaryotic and bacterial transcript processing, lifespan and decay. In the eukaryote animal hosts, mRNA transcripts generally have a half-life of many hours (Sharova et al. 2009; Yang et al. 2003) and are stabilised by the addition of a long tail of adenines (poly(A) tails; ~ 250 long) at the 3′ end of the transcript (Dendooven et al. 2020; Perez-Ortin et al. 2013; Westermann et al. 2012). These relatively stable mRNAs with long poly(A) tails can be easily captured and enriched away from rRNAs by the use of oligo (dT) primers, to generate libraries for sequencing; this is standard practice for eukaryote mRNA-Seq. In contrast, bacterial symbiont mRNAs generally have a much shorter half-life of only a few minutes on average (Selinger et al. 2003) and are transiently polyadenylated with short poly(A) tails (< 50 As) (Dendooven et al. 2020; Westermann et al. 2012) that tag the transcript for degradation, rather than for stabilisation (Dreyfus and Regnier 2002). Thus, the most common choice for rRNA depletion in bacterial studies is to use a subtractive hybridisation method via commercial kits (see Petrova et al. (2017) for comparison of different kits) before RNA-Seq analyses.

These features suggest that holobiont transcriptome analyses may require different methods to capture mRNAs from eukaryote and bacterial partners before RNA-Seq. In studies of marine sponges, well recognised as exemplar animal-bacterial holobionts (Hentschel et al. 2012; O’Brien et al. 2020; Steinert et al. 2020; Webster and Thomas 2016), numerous strategies have been used to try and capture transcriptomes from both host and symbionts. For example, to reconstruct metabolic networks linking the host sponge *Cymbastela concentrica*, a diatom and three proteobacterial symbionts, Moitinho-Silva et al. (2017) combined a eukaryote poly(A) capture with a separate bacterial rRNA depletion method. The need to treat eukaryotic and bacterial RNA separately is labour- and cost-intensive. Thus, a single method that obtains sufficiently accurate mRNA representation from host and microbe is preferred. To this end, both sponge host and bacterial symbiont transcriptomes were obtained from the giant barrel sponge *Xestospongia muta* holobiont by applying only a eukaryote rRNA depletion step (Fiore et al. 2015); it appears in that study that bacterial rRNAs were retained, but there is no analysis reported as to whether these interfered with the depth of mRNA reads acquired. In contrast, transcriptomes of both the sponge *Vaceletia* sp. and its bacterial symbionts appear to have been assessed by using only poly(A) capture method (Germer et al. 2017). Based on the differences in eukaryotic and bacterial mRNA stability and processing described above, an oligo (dT) enrichment method may be expected to capture only a portion of the bacterial mRNAs, but there appears to be remarkably little empirical evidence to support or refute this expectation.

Here, we provide, to the best of our knowledge for the first time, an empirical comparison of the completeness of transcriptomes captured by rRNA depletion compared to poly(A) capture RNA-Seq methods. Accurate determination of the fraction of expressed eukaryote and bacterial genes that are captured by poly(A) enrichment compared to rRNA depletion methods requires that transcripts can be mapped back to assembled genomes of all partners. When this is not possible because genomes are not available for both host and symbionts, it is unclear to what extent the transcriptomes represent an accurate picture of the contributions of various partners in the symbiosis. To quantitatively compare the efficacy of Poly(A)-RNA-Seq versus rRNA depletion-RNA-Seq for the capture of both animal host and bacterial symbiont transcriptomes, here, we apply both strategies to an analysis of gene expression in the demosponge *Amphimedon queenslandica* holobiont. *A. queenslandica* hosts a low diversity microbiome that is dominated by three proteobacterial symbionts (*AqS1*, *AqS2* and *AqS3*) through most of the life cycle (Fieth et al. 2016), and that play a critical role in larval settlement (Song et al. 2021). The genomes of both the host sponge (Fernandez-Valverde et al. 2015; Srivastava et al. 2010) and of the three primary symbionts have been assembled and annotated (Gauthier et al. 2016; Xiang 2021). These foundational genomic resources allow us to align transcript reads precisely to their associated genomes, making the *A. queenslandica* holobiont a useful system to compare the power of the two different strategies for RNA-seq library construction.

## Materials and Methods

### Sample Collection and Sequencing

Adult *Amphimedon queenslandica* were collected from Heron Island Reef, Great Barrier Reef, Queensland, Australia (Latitude − 23.44, Longitude 151.92) as described previously (Leys et al. 2008). Six separate tissue biopsies, each approximately 3 cm^3^, were sampled immediately after collection as a source of RNA for this study. To increase the proportional representation of the low abundance bacterial symbionts in the biopsies, bacteria were enriched by a series of centrifugation and filtration steps following the protocol of Thomas et al. (2010). To assess the quality of the bacterial cell enrichments, a small fraction of these enrichment samples were stained with 4′,6-diamidino-2-phenylindole, dihydrochloride (DAPI) following the manufacturer’s protocol and visualised by fluorescence microscopy. This allowed us to visually confirm that the enrichment process had indeed been successful in increasing the bacterial:sponge cell ratio.

Total RNA was extracted separately from the six replicated biopsies, after bacterial cell enrichment, using TRIzol (Sigma-Aldrich) and DNA was removed with deoxyribonuclease I (Invitrogen), both following the manufacturer’s protocol. RNA was quantified by Qbit^®^ 2.0 Fluorimeter (Invitrogen), and quality was assessed by 1% TAE agarose gel electrophoresis. Illumina libraries were constructed separately from each of the six total RNAs using an Illumina TruSeq Stranded mRNA Library Prep Kit.

Three of the samples were subjected to the standard TruSeq Stranded mRNA-seq workflow, using the poly-A pulldown and standard protocol with 1 µg total RNA as input. The other three samples (2.5 µg total RNA each) were first rRNA-depleted using the Ribo-Zero Gold ribosomal RNA Removal Kit (Epidemiology, supplied by Illumina; https://sapac.illumina.com/products/by-type/molecular-biology-reagents/ribo-zero-gold-rrna-removal-epidemiology.html), which removes animal, Gram-positive and Gram-negative bacterial cytoplasmic and mitochondrial rRNA. One hundred nanograms of the depleted RNA was then used as input to the mRNA-seq protocol, but omitting the poly-A pulldown and instead of starting at the RNA fragmentation step.

From the fragmentation step onwards, the TruSeq protocol was followed for all six samples, using random hexamer priming for the generation of cDNA. We used 12 PCR cycles for the amplification. Each library was sequenced on the Illumina NextSeq500 platform in four runs. Both library preparation and sequencing were performed at Ramaciotti Centre for Genomics, Sydney, Australia. Thus, all six samples were treated identically throughout except for their random assignment to either poly(A) capture or rRNA depletion.

### Read Processing and Alignment

Raw paired-end sequences of 75 base pairs (bp) in length were processed using Trimmomatic (version 0.36) (Bolger et al. 2014) to crop the first 10 bp of each read, and to trim the reads using a 4 bp sliding window and an average quality threshold of 20. Both unpaired reads and resulting reads smaller than 60 bp were discarded. The remaining quality-filtered, paired-end reads were aligned to the *A. queenslandica*, *AqS1*, *AqS2* and *AqS3* genomes (Fernandez-Valverde et al. 2015; Srivastava et al. 2010; Gauthier et al. 2016; Xiang 2021) using HISAT2 (version 2.0.5) with default parameters (Pertea et al. 2016). Both unaligned reads, and reads that aligned to more than one of these four genomes, were discarded. The read mapping bam file of each sample was split into four bam files based on the scaffold ID of each species by SAMtools (version 1.3) (Li et al. 2009).

### Comparative Assessment of Gene Depth and Gene Coverage

The number of reads that aligned to the sponge or bacterial protein-coding sequences (CDS) was counted by htseq-count (v. 0.11.2.) from the HTSeq framework, using default parameters except for –stranded = reverse (Anders et al. 2015). These aligned reads were then converted to transcript counts per gene as a measure of gene depth. In addition, for each genome, gene coverage was estimated as the percentage of genes that were represented in the RNA-Seq data (that is, expressed gene number divided by total gene number). These two measures were compared within and between libraries prepared by each of the two different methods, and with a previously generated adult transcriptome to provide further context, as described below.

### Comparison with an RNA-Seq Dataset Generated Previously from Non-Bacterial-Enriched Sponge Sample

To specifically assess the effect of bacterial enrichment on the ability to recover holobiont transcriptomes, we compared the data generated in this study (see above) with previously published deep RNA-Seq data (Fernandez-Valverde et al. 2015). The latter was a stranded Poly(A)-RNA library derived from a single adult sponge with no bacterial enrichment step and was not replicated. We directly compared results from this published data with that of bacterial-enriched Poly(A)-RNA-Seq data generated in the current study. To do so, the raw paired-end reads were filtered by Trimmomatic (parameters: SLIDINGWINDOW:4:15 MINLEN:60 HEADCROP:7) (version 0.36) (Bolger et al. 2014). The remaining high-quality pair-end reads were aligned to the holobiont genomes as described above. Gene counts and gene coverage were also calculated from these data as described above.

### Comparison of Gene Function Among Different Data Sets

To investigate the possibility of functional biases resulting from differential capture of transcripts, Gene Ontology (GO) analyses were performed for the genes captured by the three different library preparation methods, rRNA-depleted-RNA-Seq, Poly(A)-RNA-Seq and bacterial-unenriched Poly(A)-RNA-Seq. GO term annotations of the holobiont gene models were performed using Blast2GO (version 5.2.4) (Conesa and Götz 2008), and the number of genes assigned to each annotated GO term was calculated for each species. Expressed CDS in each sample were identified as those to which at least one read pair mapped and expressed GO terms were scored when there was at least one gene expressed that could be attributed to the GO term. To identify any differences in the GO functions among the different RNA-Seq libraries, the expressed GO percentages were estimated in each sample for the sponge host and for each of the three bacterial symbionts, separately. That is, for each of the four species, the number of expressed GO terms in a given sample was divided by the total number of GO terms annotated in that species; the three replicated samples per RNA-Seq dataset were averaged. As several genes would likely be annotated with the same GO function, for each expressed GO term, the number and proportion of expressed genes were also calculated for each RNA-Seq dataset; the three replicated samples per RNA-Seq dataset were averaged.

## Results

### Sequencing and Alignment Profile

We generated 388 and 403 million reads for the three replicate rRNA-depleted-RNA-Seq samples (a, b, c) and the three replicate Poly(A)-RNA-Seq samples (d, e, f), respectively. After filtering low-quality reads with Trimmomatic (Bolger et al. 2014), more than 86% of reads (~ 336 and ~ 354 million reads for rRNA-depleted and Poly(A) samples, respectively) remained for subsequent analysis. For the previously published bacterial-unenriched Poly(A)-RNA-Seq transcriptome (Fernandez-Valverde et al. 2015), which was unreplicated, 341 million reads were generated, and 320 million of these reads remained after the quality filter step. All these high-quality reads were then aligned to updated versions of the genomes of *A. queenslandica* and its proteobacterial symbionts *AqS1*, *AqS2* and *AqS3* (Xiang 2021) by HISAT2 (version 2.0.5) (Pertea et al. 2016). On average, 69.3% rRNA-depleted-RNA-Seq reads, 81.2% Poly(A)-RNA-Seq reads and 63.1% bacterial-unenriched Poly(A)-RNA-Seq reads mapped to the four genomes. Of these mapped reads, 4.8% rRNA-depleted-RNA-Seq reads, 0.1% Poly(A)-RNA-Seq and few bacterial-unenriched Poly(A)-RNA-Seq reads mapped to more than one genome and were discarded because they could not be unambiguously assigned. A total of 222 million mapped rRNA-depleted-RNA-Seq reads, 287 million mapped Poly(A)-RNA-Seq reads and 51 million mapped bacterial-unenriched Poly(A)-RNA-Seq reads remained for further analysis.

**Fig. 1:**
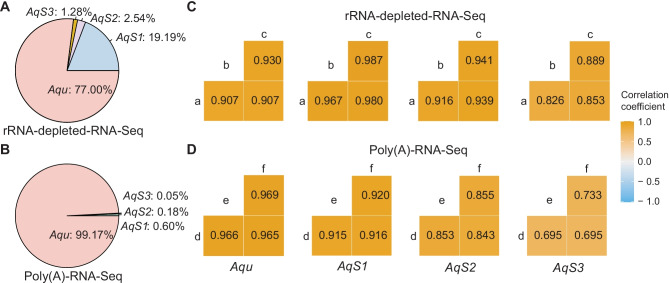
Comparison between two different RNA-Seq methods in capturing transcriptomes of the *A. queenslandica* holobiont. **A** and **B** Taxonomic distribution of reads, expressed as percent of reads aligned to *A. queenslandica* (*Aqu*) and its proteobacterial symbionts *AqS1*, *AqS2* and *AqS3* for rRNA-depleted-RNA-Seq (A) and Poly(A)-RNA-Seq (B) libraries. **C** and **D** Correlation of expressed genes between three biological replicates for rRNA-depleted-RNA-Seq (C, replicates a–c) and Poly(A)-RNA-Seq (D, replicates d–f) libraries

For the bacterial-enriched rRNA-depleted-RNA-Seq libraries, 77% of the mapped reads were attributed to the sponge host *Aq*, while 19.2%, 2.54% and 1.3% were attributed to *AqS1*, *AqS2* and *AqS3*, respectively (Fig. 1A). By comparison, for the bacterial-enriched Poly(A)-RNA-Seq libraries, almost all the aligned reads (99.2%) were attributed to the sponge host; only 0.6%, 0.2% and 0.1% reads were attributed to bacterial symbiont genomes *AqS1*, *AqS2* and *AqS3*, respectively (Fig. 1B). For the bacterial-unenriched Poly(A)-RNA-Seq library, 99.9% of mapped reads were attributed to the sponge host, and only 0.03% to the three symbiotic bacteria in total.

### Correlation Between Samples

The reads aligned to each gene were considered a measure of gene expression. Correlation of the gene expression between the technical triplicates was estimated by gene read counts (gene depth) between individuals (biological replicates) within each experimental group. All read counts were included in calculating the Spearman's correlation efficiencies. For the host sponge *A. queenslandica*, the gene expression correlation was strong among the triplicates from both rRNA-depleted- and Poly(A)-RNA-Seq groups, with Spearman’s correlation efficiencies ranging from 0.907 to 0.969 (Fig. 1C, D). For the three symbiotic bacteria, the correlation of gene expression varied between the biological replicates from each experimental group. For the rRNA-depleted-RNA-Seq data, the Spearman's correlation efficiencies of *AqS1* biological replicates ranged from 0.967 to 0.987, with *AqS2* from 0.916 to 0.941, and *AqS3* from 0.826 to 0.889. These were higher than those correlation efficiencies of the Poly(A)-RNA-Seq replicates, with *AqS1* ranged from 0.915 to 0.920, *AqS2* from 0.843 to 0.855 and *AqS3* from 0.695 to 0.733. Overall, the strong correlations between the biological replicates from each experimental group suggested the gene expression profiles of the four species in the *A. queenslandica* holobiont were comparable within the rRNA-depleted- and the Poly(A)-RNA-Seq samples.

### Gene Coverage

**Fig. 2:**
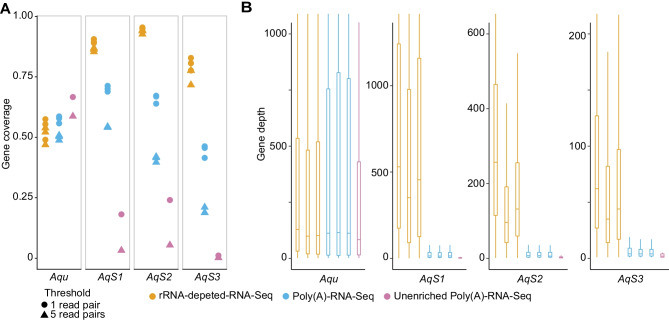
Gene coverage and depth of rRNA-depleted-RNA-Seq, Poly(A)-RNA-Seq and unenriched Poly(A)-RNA-Seq transcriptomes of the *A. queenslandica* holobiont. **A** Gene coverage, expressed as percent of genes to which reads aligned to *A. queenslandica* (*Aqu*) and its proteobacterial symbionts *AqS1*, *AqS2* and *AqS3*. Shown are results for 1 (dots) and 5 (triangles) reads mapped to CDS thresholds for the three biological replicate libraries (rRNA-depleted-RNA-Seq and Poly(A)-RNA-Seq) and single bacterial-unenriched Poly(A)-RNA-Seq library. **B** Gene depth, expressed as boxplot of the number of reads mapped to each expressed gene

Transcript quantification is one of the most common applications of RNA-Seq; it estimates the gene expression levels based on the number of reads mapped to each gene. This approach is widely used to identify differential expression (DE) of genes between different treatments and contexts (Conesa et al. 2016). Many DE software packages filter genes with < 5 reads before identifying DE genes because of both biological and statistical concerns, removing genes with low read counts prior to downstream analysis (Chen et al. 2016). To better understand the influence of RNA-Seq library preparation method for capture of both animal host and bacterial symbiont transcriptomes, we compared the percentage of genes to which RNA-Seq reads were mapped across the different methods (Fig. 2A). Specifically, we filtered the genes with two thresholds (5 and 1 read pairs) and then calculated the gene coverage.

When we used the minimal threshold of 1 read pairs mapped, the percent of *A. queenslandica* genes covered by the rRNA-depleted-RNA-Seq data (53.93%) was very similar to that of the Poly(A)-RNA-Seq data (57.38%) (two-tailed *t*-test, *p*-value 0.308), and both of these were lower than the bacterial unenriched-Poly(A)-RNA-Seq data (66.62%) (Fig. 2A). For the bacterial symbiont genomes, on average, 89.70% (± 0.0082) *AqS1* genes, 95.11% (± 0.0029) *AqS2* genes and 80.36% (± 0.0260) *AqS3* genes were captured by rRNA-depleted-RNA-Seq libraries. These gene coverages were much higher than those captured by Poly(A)-RNA-Seq libraries, which, on average, were 70.07% (± 0.0119) for *AqS1*, 65.99% (± 0.0181) for *AqS2* and 44.46% (± 0.0259) for *AqS3* (one-tailed *t*-test, *p*-values were 2.54e-05 in *AqS1*, 4.98e-04 in *AqS2* and 3.56e-05 in *AqS3*). The coverages also were higher than that of the bacterial unenriched-Poly(A)-RNA-Seq samples, which were 18.14% in *AqS1*, 24.06% in *AqS2* and 1.13% in *AqS3*. This gene coverage comparison indicated that rRNA-depleted-RNA-Seq and Poly(A)-RNA-Seq could capture the animal host transcriptomes at the same level, but rRNA-depleted-RNA-Seq was better at capturing the bacterial symbiont transcriptomes. The unenriched-Poly(A)-RNA-Seq was less likely to comprehensively capture bacterial symbiont transcriptomes compared to the rRNA-depleted-RNA-Seq and Poly(A)-RNA-Seq.

When the threshold was raised to at least 5 read pairs per gene, on average, 50.94% and 49.92% *A. queenslandica* genes were covered in the rRNA-depleted-RNA-Seq data and Poly(A)-RNA-Seq data, respectively, with 58.66% of the genes represented in bacterial unenriched-Poly(A)-RNA-Seq data (Fig. 2A). These values are consistent with the estimated proportion of genes in the genome that are expressed in adult *A. queenslandica* (Fernandez-Valverde et al. 2015). For the symbiont bacterial genes, on average, 86.23% (± 0.0079) *AqS1* genes, 93.65% (± 0.0087) *AqS2* genes and 75.63% (± 0.0344) *AqS3* genes were captured in the rRNA-depleted-RNA-Seq data. By comparison, in the Poly(A)-RNA-Seq data, on average, only 54.22% (± 0.0016) *AqS1* genes, 41.07% (± 0.0123) *AqS2* genes and 19.57% (± 0.0130) *AqS3* genes were captured. In the bacterial unenriched-Poly(A)-RNA-Seq samples, only 3.22% *AqS1* genes, 5.43% *AqS2* genes and 0.20% *AqS3* genes were captured. These gene coverages indicate that rRNA-depleted-RNA-Seq captured a similar proportion of sponge genes and a higher proportion of bacterial genes than Poly(A)-RNA-Seq. For the unenriched-Poly(A)-RNA-Seq sample, the coverage was representative only for the sponge transcriptome but not for the symbiotic bacterial transcriptomes.

### Distribution of Read Depth in Gene Coding Regions

Sequence depth is a crucial measure of the robustness of RNA-Seq data. We calculated the gene depth by counts of aligned reads per gene and compared the gene depth distribution of the rRNA-depleted-, Poly(A)- and bacterial unenriched-Poly(A)-RNA-Seq samples (Fig. 2B). For the host sponge *A. queenslandica*, on average, the median gene depths were 110 (± 15.95) and 113 (± 1.73) reads per gene for the rRNA-depleted- and Poly(A)-RNA-Seq libraries, respectively, and 83 for the bacterial unenriched-Poly(A)-RNA-Seq library. For each bacterial symbiont, the rRNA-depleted-RNA-Seq data showed the highest average gene depths, which were higher than these of the Poly(A)-RNA-Seq data and bacterial unenriched-Poly(A)-RNA-Seq data. On average, the rRNA-depleted-RNA-Seq median gene depths were 446 (± 89.37) in *AqS1*, 162 (± 84.50) in *AqS2* and 47 (± 13.75) in *AqS3*; the Poly(A)-RNA-Seq median gene depths were 14 (± 0.58) in *AqS1*, 7 (± 0.58) in *AqS2* and 4 (± 0) in *AqS3* (Fig. 2B). The median gene depth for all the three primary symbiotic bacteria was 2 for the bacterial unenriched-Poly(A)-RNA-Seq sample. The similar read depth distributions of *A. queenslandica* for rRNA-depleted- and Poly(A)-RNA-Seq samples implied that there was no noteworthy gene depth difference among the host sponge transcriptome captured by both methods, but sponge transcript depths were lower than obtained by bacterial unenriched-Poly(A)-RNA-Seq (Fig. 2B). The symbiont gene depths were much higher in the rRNA-depleted-RNA-Seq data compared to both the Poly(A)-RNA-Seq and the bacterial unenriched-Poly(A)-RNA-Seq data.

### Comparison of the Expressed Biological Functions in the Different RNA-Seq Data Sets

A first step towards understanding gene functions in host and symbionts is to annotate expressed and enriched genes in RNA-Seq data sets using Gene Ontology (GO) (Ashburner et al. 2000). GO analyses were performed and compared between rRNA-depleted-RNA-Seq, Poly(A)-RNA-Seq and bacterial unenriched Poly(A)-RNA-Seq using Blast2GO (version 5.2.4) (Conesa and Götz 2008) to determine if any of the RNA-Seq approaches resulted in a bias in the types of genes captured in the transcriptomes. In total, 28,570 *A. queenslandica* genes, 2417 *AqS1* genes, 1193 *AqS2* genes and 1977 *AqS3* genes were assigned to 6357, 1317, 904 and 1030 GO terms, respectively.

**Fig. 3:**
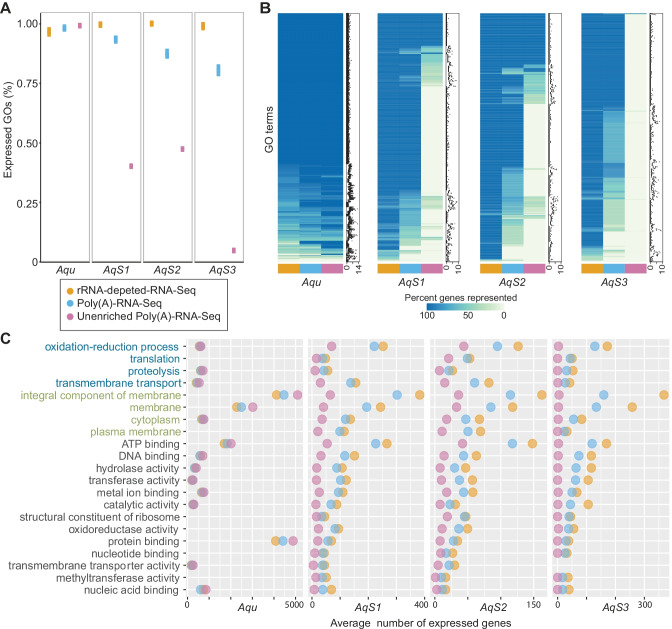
Gene ontology analyses of transcriptomes generated by the RNA-Seq data sets of the partners in the *A. queenslandica* holobiont. **A** Percent of GO terms identified from expressed genes present in rRNA-depleted-RNA-Seq, Poly(A)-RNA-Seq and unenriched Poly(A)-RNA-Seq datasets with at least 1 read pair per CDS compared to genome GO terms. **B** Heatmaps presenting the average percent of genes represented by each GO term; adjacent dot plots show total gene number attributed to each GO term with the *x*-axis on a log2 scale. **C** Average number of expressed genes for the 30 GO terms that are most differentially represented between the different RNA-Seq datasets. Blue lettering, biological processes; green lettering, cellular components; black lettering, molecular functions. See Supplementary Table S1 for the number and proportion of expressed *A. queenslandica*, *AqS1*, *AqS2* and *AqS3* genes in each GO category

To identify any differences in GO functional annotations among the different RNA-Seq libraries, the percentage of expressed GO terms in each sample was calculated for each species. That is, for a given species, if any gene in the genome that had been assigned to a particular GO term was expressed with at least 1 read pair in a given sample, that GO term was considered an expressed GO term. The expressed GO percentage was estimated as the percent of total GO terms for the genome that were represented in the RNA-Seq data, with replicates averaged (Fig. 3A and Supplementary Table S1). For *A. queenslandica*, almost all the GO terms were present in the rRNA-depleted-RNA-Seq data (95.88% ± 0.0085), Poly(A)-RNA-Seq data (97.43% ± 0.0036) and bacterial unenriched-Poly(A)-RNA-Seq data (98.47%). For the bacterial symbionts, the expressed GO term percentages were 98.96% ± 0.002 in *AqS1*, 99.34% ± 0.001 in *AqS2* and 98.32% ± 0.006 in *AqS3* for the rRNA-depleted-RNA-Seq samples (Fig. 3A). These percentages of expressed GO terms in rRNA-depleted-RNA-Seq samples were higher than in Poly(A)-RNA-Seq samples, which was 92.63% ± 0.006 in *AqS1*, 86.32% ± 0.01 in *AqS2* and 79.58% ± 0.014 in *AqS3* (one-tailed *t*-test, *p*-values were 3.68e-04 in *AqS1*, 9.37e-04 in *AqS2* and 1.91e-04 in *AqS3*); and also higher than that of bacterial unenriched-Poly(A)-RNA-Seq sample, which was 38.72% in *AqS1*, 46.02% in *AqS2* and 2.91% in *AqS3* (Fig. 3A). These GO term expression percentages indicate that the different libraries capture a similar functional transcriptional profile of the host sponge *A. queenslandica*. In contrast, while rRNA-depleted-RNA-Seq provides a near-complete functional representation of the symbiotic bacteria, this is not true for the Poly(A)-RNA-Seq and bacterial unenriched-Poly(A)-RNA-Seq.

GO functional profiles of expressed and over-represented (enriched) genes in the three RNA-Seq data sets, with replicates averaged, were compared to determine if GO categories were equally represented in the different types of RNA-Seq libraries (Fig. 3B and Supplementary Table S1). This approach revealed differences in the representation of specific GO functions in each of the libraries. For the sponge *A. queenslandica*, on average, the expressed gene percentage of each GO term was higher in the bacterial unenriched-Poly(A)-RNA-Seq data (93.64%) compared to the rRNA-depleted-RNA-Seq data (86.57%) and the Poly(A)-RNA-Seq data (90.62%). For each bacterial symbiont, the rRNA-depleted-RNA-Seq data had the highest GO term expressed gene percentages compared to the Poly(A)-RNA-Seq data and bacterial unenriched-Poly(A)-RNA-Seq data (Fig. 3B). The rRNA-depleted-RNA-Seq average GO term expressed gene percentages were similar, 95.57, 98.62% and 95.62% for *AqS1*, *AqS2* and *AqS3*, respectively. In contrast, the average GO term expressed gene percentages of Poly(A)-RNA-Seq and bacterial unenriched-Poly(A)-RNA-Seq for the three symbionts varied considerably. For the Poly(A)-RNA-Seq, the average GO term expressed gene percentages decreased from 84.49% in *AqS1* to 77.45% in *AqS2* and 67.85% in *AqS3*. For the bacterial unenriched-Poly(A)-RNA-Seq, this percentage decreased from 21.88% in *AqS1* and 31.27% in *AqS2* to 0.43% in *AqS3* (Fig. 3B). These GO term percentages suggest that for the symbionts, the Poly(A)-RNA-Seq method produces bias in the functional types of genes that are captured.

We then used pairwise comparisons of expressed gene numbers in each of the RNA-Seq data sets to identify which functional groups of genes, as indicated by GO terms, were most differently captured by the different library types. For each pairwise comparison, the most differentially represented 30 GO terms were identified by the biggest differences of the expressed gene numbers. The most differentially represented GO terms included four biological process GOs (oxidation–reduction process, proteolysis, transmembrane transport and translation), four cellular component GOs (integral component of membrane, membrane, cytoplasm and plasma membrane) and 13 molecular function GOs (e.g. catalytic activity, hydrolase activity, transferase activity, ATP binding and DNA binding) (Fig. 3C). Overall, compared to unenriched-Poly(A)-RNA data, the rRNA-depleted- and Poly(A)-RNA data sets under-represented *A. queenslandica* genes involved in integral component of membrane, membrane, ATP binding and protein binding (Fig. 3C). For the three symbiotic bacteria, compared to the rRNA-depleted RNA data, the Poly(A)- and bacterial unenriched-Poly(A)-RNA data most underrepresented genes involved in oxidation–reduction, transmembrane transport, integral component of membrane and ATP binding (Fig. 3C).

## Discussion

### Transient Polyadenylation in Bacteria Reduces mRNA Capture in Poly(A)-RNA-Seq

Sequencing depth and coverage are central to assessing the efficacy of RNA-Seq (Sims et al. 2014). In this transcriptome analysis of the four dominant partners in the marine sponge *A. queenslandica* holobiont, we sought to identify which of the RNA-Seq approaches that we compared yielded the highest depth and coverage for genes expressed in both the sponge host and its three proteobacterial symbionts. Under conditions used in this study — i.e. libraries from bacterial-enriched cell preparations from adult sponges — we found that both rRNA-depleted- and Poly(A)-RNA-Seq capture sponge host transcripts at similar levels with at least 5 read pairs aligned (on average, 50.94% and 49.92% of genes in the genome). In contrast, only the rRNA-depleted-RNA-Seq captured a large proportion of the expressed genes in the symbionts (86.23% *AqS1*, 93.65% *AqS2* and 75.63% *AqS3* of genes per genome), compared to much smaller proportions obtained by Poly(A)-RNA-Seq (54.22% *AqS1*; 41.07% *AqS2*; 19.57% *AqS3* of genes per genome). This difference potentially reflects the different capacities of these two RNA-Seq approaches to efficiently capture bacterial mRNAs, which are relatively unstable (Selinger et al. 2003) and are polyadenylated just prior to degradation (Dreyfus and Regnier 2002). The Poly(A)-RNA approach captures a smaller number of bacterial mRNAs, because it can only capture those in the very transient state of polyadenylation coincidentally at the exact time of sample fixation.

Interestingly, however, the subset of bacterial mRNAs captured by Poly(A)-RNA-Seq method does not appear to provide a random sample of the bacterial transcriptome profile. If random, we expect all GO categories to reduce proportionally with the reduction in transcript coverage observed when comparing Poly(A)-RNA-Seq and rRNA-depleted-RNA-Seq libraries. Although many GO terms are shared between libraries (*AqS1*, 502; *AqS2*, 396, and *AqS3*, 29), we find there are 54 *AqS1*, 50 *AqS2* and 89 *AqS3* GO terms that are present only in rRNA-depleted-RNA-Seq transcriptomes. This bias appears not to support bacterial polyadenylation being a ubiquitous and random post-transcriptional modification (Maes et al. 2017), with poly(A) polymerases having no mRNA sequence specificity (Haugel-Nielsen et al. 1996). The differences between the detected and undetected GO terms in the poly(A)-RNA samples in this study suggest that mRNA turnover rate in *AqS1*, *AqS2* and *AqS3* may be gene-dependent. This observation is akin to that observed in other bacteria where variable mRNAs decay rates (Hui et al. 2014) could yield biased subsets of bacterial mRNAs captured by poly(A)-RNA-Seq. For instance, in *Escherichia coli*, RNAs from only 110 genomic regions were detected as being polyadenylated; other polyadenylated RNAs appear to degrade too rapidly to be detected (Maes et al. 2017). Bacterial mRNA turnover is modulated by ribonucleases (RNases), RNA binding proteins and small noncoding RNAs (sRNA) (Anderson and Dunman 2009) and could be facilitated by poly(A) polymerase (Hajnsdorf and Kaberdin 2018). These regulatory processes can play a vital role in bacterial stress adaptation, cell growth and virulence factor production (Anderson and Dunman 2009; Li et al. 2017). Interestingly, the GO categories captured in this study by rRNA depletion but not by poly(A) capture include processes related to stress adaptation and growth.

### rRNA-Depleted-RNA-Seq Best Captures the Holobiont Transcriptome

We found that the rRNA-depleted-RNA-Seq simultaneously captures a high proportion of the expressed genes in the sponge host and in the bacterial symbionts, which is in contrast to the Poly(A)-based RNA-Seq approach that we tested. Importantly, there was not a marked difference between sponge host transcriptome representation in rRNA-depleted- and Poly(A)-RNA-Seq data sets that were derived from bacterial cell enrichment. The similar output from both rRNA-depleted- and Poly(A)-RNA-Seq datasets indicate that (i) a sufficient number of sponge cells are captured using this bacterial cell enrichment method to generate highly representative RNA-Seq libraries and (ii) there is no substantial loss of host transcript abundance or representation in the rRNA-depleted-RNA-Seq dataset. In contrast, all three bacterial symbiont transcriptomes are significantly better covered using the rRNA-depleted-RNA-Seq approach. This high bacterial gene coverage in the rRNA-depleted-RNA-Seq libraries is similar to the human-pathogen system where an rRNA-depleted-RNA-Seq method captured 88% of the *Haemophilus influenzae* genes from in vitro human bronchial epithelium; the human transcriptome was simultaneously captured (Baddal et al. 2015). In addition, rRNA-depleted-RNA-Seq also improves detection of low abundance transcripts (Kim et al. 2019; Petrova et al. 2017) and noncoding RNA (Westermann et al. 2016), which could reveal a more complex host-symbiont transcriptome profile.

Despite the ability of rRNA-depleted-RNA-Seq to capture a better representation of bacterial transcripts, our study also demonstrates that this approach may not obtain a near-complete representation of transcripts from the less abundant bacterial members of the holobiont. *AqS1* alone comprises well over 50% of the bacterial community (Fieth et al. 2016). While its transcriptome appears to be well-represented, the least abundant of major symbionts, *AqS3* (3.5% of the community), is less well representative (gene depth is similar to *AqS1* but gene coverage is lower — 80.4% vs 89.7%), assuming this difference is not related to biological differences (i.e. proportion of the genome expressed). Increasing the amount of input RNA from bacterial enrichments, and/or increasing the number of reads generated, could relatively easily increase the amount of available sequence data to be mapped to the genomes of less abundant members of the holobiont. This will increase the transcript representation of numerically minor members of the holobiont.

## Conclusion

We provide here the first, to the best of our knowledge, quantitative and qualitative comparison of RNA-Seq methods that address the high proportion of rRNAs in both eukaryotic and prokaryotic transcriptomes by either selecting for polyadenylated mRNAs or depleting rRNAs. In doing so, we empirically demonstrate that the latter approach — depleting rRNAs prior to construction of an RNA-Seq library — can be applied to a marine animal holobiont to successfully achieve simultaneous and quite comprehensive capture of transcripts of both the animal host and its bacterial symbionts. Although selection for polyadenylated mRNAs, which is the approach routinely used for animal transcriptomes, does indeed capture some of the bacterial symbiont transcripts, it does so in a biased manner that renders it unsuitable for symbiosis studies where the goal is to study gene expression in both host and symbionts. Instead, depleting rRNA in both host and symbionts in a single step provides a time- and cost-efficient approach that can proceed through to simultaneous capture of transcriptomes from all partners using the same biological samples. The approach should be relatively easily applied to any holobiont comprising a eukaryotic host and bacterial symbionts.

## Supplementary Information

Below is the link to the electronic supplementary material.

Supplementary file1 (XLSX 814 KB)

## Data Availability

Genomes of the marine sponge *A. queenslandica* and of the three proteobacterial symbionts, *AqS1*, *AqS2* and *AqS3*, used in this study are available at NCBI under Bioproject accession PRJNA668660t. RNASeq datasets generated in this study are available at NCBI under accession GSE205425.
